# Overview of the Use of Murine Models in Leukemia and Lymphoma Research

**DOI:** 10.3389/fonc.2017.00022

**Published:** 2017-02-20

**Authors:** Rebecca Kohnken, Pierluigi Porcu, Anjali Mishra

**Affiliations:** ^1^Department of Veterinary Biosciences, College of Veterinary Medicine, The Ohio State University, Columbus, OH, USA; ^2^Comprehensive Cancer Center, James Cancer Hospital and Solove Research Institute, The Ohio State University, Columbus, OH, USA; ^3^Division of Hematology, Department of Internal Medicine, The Ohio State University, Columbus, OH, USA; ^4^Division of Dermatology, Department of Internal Medicine, The Ohio State University, Columbus, OH, USA

**Keywords:** leukemia, lymphoma, mouse models, hematologic neoplasms, mouse genetic models

## Abstract

Murine models have been adopted as a significant and powerful tool in the study of cancer. The applications of murine models of cancer are numerous: mechanism discovery, oncogenesis, molecular genetics, microenvironment, metastasis, and therapeutic efficacy. Leukemias and lymphomas are a group of highly heterogeneous hematologic malignancies that affect people of all ages and ethnicities. Leukemia and lymphoma arise from hematopoietic and immune cells and usually spread widely throughout the body. The liquid nature of many of these malignancies, as well as the complex microenvironment from which they arise and their multifaceted genetic basis, has added to the difficulty in generating appropriate and translational models to study them. Murine models of leukemia and lymphoma have made substantial contributions to our understanding of the pathobiology of these disorders in humans. However, while there are many advantages to these models, limitations remain. In this review, we discuss the mouse as a model to study leukemia and lymphoma, and the importance of choosing the correct methodology. Specific examples of murine models of leukemias and lymphomas are provided, with particular attention to those that are highly translational to their human counterpart. Finally, future applications of murine models and potential for better models are discussed.

## Introduction

In “The Principles of Humane Experimental Technique” published in 1959, William Russell and Rex Burch proposed that every effort should be made to replace experimental animals with non-sentient alternatives, to reduce the number of animals used, and to refine laboratory procedures with the aim of causing minimal pain and distress to research animals (1, 2). Now known as the “3Rs,” these principles have been since adapted and developed for modern biomedical research uses.

A key goal of biomedical research is to provide clinicians with advanced knowledge to predict disease pathology and select appropriate treatment. While much of the work done in research is accomplished *in vitro* or *in silico*, the predictive value of these data is ultimately limited by the complexity of whole-organism systems. The critical advantage of animal models is that it can be used to test relationships and mechanisms under controlled experimental conditions, which can then be translated to predict human clinical outcomes. The challenge falls to researchers to anticipate distinct differences between cellular behavior *in vitro* in contrast to *in vivo*, and additionally the inherent differences between species to generate data that is translatable to the clinic.

Mouse models of human disease are highly valuable in biomedical research, but only with appropriate validation and careful consideration of the compatibility of the data with human disease (3). Validation involves determining the extent to which the mouse phenotype mimics the clinical characteristics of the human illness (3). For the study of cancer, in particular, the ideal murine model should replicate the genetic and molecular heterogeneity of tumors and involve *de novo* tumors in immune-competent mice while also mimicking clinical behavior of the human disease. These murine models should develop tumors with high penetrance and reproducibility, while also offering a mechanism of monitoring disease progression and treatment efficacy (4).

## The Mouse as a Model

Many organisms are available to research scientists, each with their advantages and disadvantages for the study of individual organ systems and disease types. Despite the wide variety of available model systems, the mouse (*Mus musculus*) is now widely considered the model organism of choice for the study of human disease. The reasons for this choice are numerous, including the relative genetic similarity of mice to humans, their small size, reliable breeding, and short life span. Mice also share many physiologic characteristics with humans and, therefore, provide similar organ system biology for the study of cardiovascular, endocrine, immune diseases, and others.

Historically, mice were used in genetic experiments as early as 1930 due to their abundant availability (5). Today, breeding inbred strains, developing models, and shipping mice for research use is a major industry. There are strains that spontaneously develop diseases of interest, as well as genetically manipulated strains that are prone to developing certain cancers, obesity, glaucoma, etc. (5). Additional models available include immunodeficient mice that are valuable for the study of cancer and certain infectious diseases, as well as being acceptable hosts for human tissue and cells. Powerful tools available to compare mouse and human genomes have allowed comprehensive genomic manipulation in the mouse to mimic human disease pathophysiology (5). For conditions lacking an accurate model, experimental approaches to humanize mice can be used to more closely mimic human disease.

For all of their advantages, there are also numerous limitations that must be considered when choosing the model and interpreting their findings. From the conception of the model, through development, data collection, and interpretation, the idiosyncrasies superimposed onto the data by the “mouse factor” must be considered. One prominent example of this problem is in the general unfamiliarity of background lesions in different mouse strains. Some spontaneous disease conditions are strain or age related; and while inbred mice are genetically extremely similar, minor differences exist and can result in variability within the study. Therefore, while the mouse is a powerful tool, it must also be viewed as an independent contributor to the research conducted upon it and as one that deserves consideration.

Concerning hematopoiesis, in mice, lymphocytes are the predominant circulating white blood cell, whereas the neutrophil predominates in humans (3, 6, 7). Importantly, extramedullary hematopoiesis in the red pulp of the spleen is physiologic in the adult mouse and can be exuberant and should not interpreted as neoplasia (6). Additionally, bone marrow in the mouse retains abundant hematopoietic components throughout life in contrast to the paucicellular marrow of adult humans. Also, ectopic thymic tissue in the mouse can be found commonly in various locations in the cervical region, and should not be confused with infiltrative neoplasia (3).

## Choosing the Correct Model

The choice of model is often the first and most important consideration in developing *in vivo* research studies. First and foremost, is the mouse the best model? It may not be. It may be that better models are available but too expensive or otherwise not practical. For modeling many diseases, particularly cancer, there is likely no significant difference between the utility of the mouse versus other laboratory species, and the decision comes down to availability and cost. Murine models are abundantly plentiful commercially, and as a standard of research are often easiest to cite and utilize previous work to develop your model.

One early and critical consideration is the selection of the strain of mouse. Inbred strains are the result of greater than 20 consecutive generations of sister–brother or parent–offspring matings. These mice will be homozygous at virtually all loci. Each strain has distinguishing characteristics as well as significant genotypic and phenotypic differences within a single strain. Often, it is acceptable and sufficient to access online databases to choose your knockout of interest, on a standard background strain, such as FVB/N, BALB/c, or C57BL/6. C57BL/6 are often utilized for mutagenesis studies and are overall the most commonly used strain in academic institutions (3). Frequent spontaneous diseases in C57BL/6 include hydrocephalus, staphylococcal dermatitis, and pulmonary proteinosis (7). BALB/c mice commonly develop myocardial degeneration and left auricular thrombosis. FVB/N mice are often used to generate transgenic animals as they have large pronuclei for gene injection and tend to be natural superovulators. This strain is prone to development of seizures and mammary hyperplasia secondary to prolactinomas (7). One hundred twenty-nine mice are often used as donors of embryonic stem cells and are prone to pulmonary proteinosis and hypocollosity (7). Aside from these common background findings, each of these strains is predisposed to developing certain spontaneous tumors, with lymphoma often being most common (7). The high incidence of leukemia and lymphoma in some mouse strains is further enhanced in immunocompromised mice—an experimental tool often used to model these diseases. Some spontaneous leukemias, in particular, can be difficult to distinguish from the disease model manipulation. It is clear that many of these conditions could confound individual studies, and therefore, the strain of mouse must be considered carefully.

## Developed Mouse Models and Their Utility

There are multiple advantages of using the mouse with regard to its genetic similarity to people, including a similar number of protein-coding genes, 40% direct gene alignment between the two species, and the finding that 99% of human genes have homologous genes in the mouse (6, 8). Advances in genomic techniques have allowed development of highly translational murine models of hematologic malignancies. Classifications of murine models of human hematologic neoplasia include spontaneous, xenograft, and genetically engineered models (Table 1).

**Table 1:** **Advantages and disadvantages of types of murine models**.

Model	Advantages	Disadvantages
Spontaneous	No/minimal manipulation needed Whole-organism system available to study disease pathogenesis	May not be accurate/translational to human disease condition Typically arises in older animals—time consuming and more costly

Xenograft (cell line-derived)	Relative simplicity High yield Rapid results Relatively inexpensive Multiple routes of administration Avoid immune rejection with immunocompromised strains (commercially available) Useful as first step investigation Useful as confirmatory for *in vitro* findings	Lack of organ/system microenvironment (except for orthotopic) Lack of immune system interaction with tumor cells Relative inability to test complex genomic interactions in a single-cell system Cell lines likely differ significantly from parental source (tumor)

Xenograft (patient-derived)	Relative simplicity High yield Rapid results Multiple routes of administration Avoid immune rejection with immunocompromised strains (commercially available) Useful as first step investigation More true representation of tumor cell biology than above Useful for investigation into efficacy of therapeutics on human tumor cells	Lack of organ/system microenvironment (except for orthotopic) Lack of immune system interaction with tumor cells Relative inability to test complex genomic interactions in a single-cell system

Humanized mice	Competent immune system to model tumor–immune interaction Can engraft cell lines, human tumor tissue, or genetically manipulated cells More true representation of tumor cell biology in a human-like system Useful for investigation of tumor pathobiology	Expensive Time consuming Generally need to establish breeding colony

Germline transgenic	Faithful alteration of gene of interest Immunocompetent mice Useful for testing tumor development Useful for testing therapeutic approaches Useful for testing chemopreventative techniques	Transgene is universally expressed in every tissue Transgene is expressed throughout embryologic development Genetically not as complex as many human tumors

Conditional transgenic	Faithful alteration of gene of interest Immunocompetent mice Targeted tissue-specific expression Targeted temporal expression Useful for testing tumor development Useful for testing therapeutic approaches Useful for testing chemopreventative techniques	Challenging technique Expensive Many mice will not carry desired genotype following crossing Genetically not as complex as many human tumors

Multi-allelic transgenics (clustered regularly interspaced short palindromic repeats-Cas9)	Multiple genes can be manipulated to more closely mimic disease complexity Ability to test cooperating mutations	Challenging technique Expensive Potential of off-target mutations

**Figure 1:**
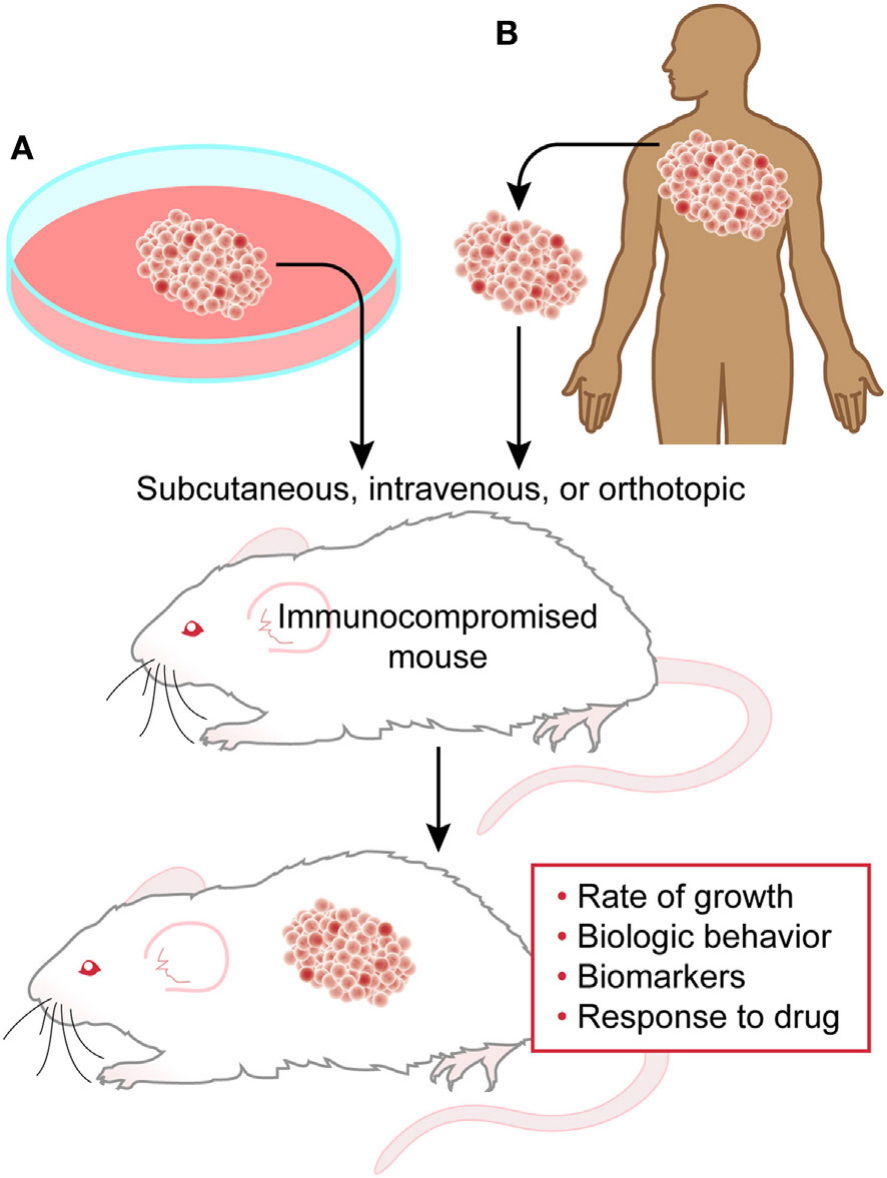
**Xenograft model**. Cell line-derived **(A)** or patient-derived **(B)** cells can be transplanted into immunocompromised mice to study several aspects of tumor biology and behavior.

Xenograft models are relatively simple, high-yield, potentially high-reward systems, which can be used to begin investigations into tumor biology. Human tumor tissue or tumor-derived cell lines are transplanted subcutaneously, intravenously, or orthotopically into the organ type of origin (Figure 1). These models are conducted in immunocompromised mice to avoid immune rejection of the human cells (9). Immunocompromised mice range from athymic nude to variations on severe combined immunodeficiency (SCID) mice. Nude mice are generated on BALB/c background utilizing a mutation in Foxn1 resulting in the lack of thymic development, and therefore thymus-derived mature T cells. Importantly, these mice still carry B cells, granulocytes, dendritic cells, and highly active NK cells. SCID mice are typically ΔPrkdc and Rag1 null, derived on a C57BL/6, C3H, or non-obese diabetic/LtSzJ backgrounds. These mutations result in the lack of functioning B and T cells with minimal to no NK activity. Certain commercially available NOD/SCID strains are also somewhat resistant to the development of spontaneous lymphoma (10). Xenograft models are highly useful in determining *in vivo* proof-of-concept from *in vitro* studies as well as examining the efficacy of therapeutics on human tumor cells. Their significant disadvantages, however, include a general lack of tumor context or microenvironment (partially improved by orthotopic models), and inability to determine the effects of immune systems on tumor growth. NSG™ mice are further immunocompromised with an IL2Rγ knockout, rendering these mice deficient in all lymphocytes, including NK cells, making these mice more receptive to engraftment. NOD/SCID mice can be “humanized” by the addition of human peripheral blood lymphocytes, bone marrow, or fetal liver and thymus into irradiated or immunodeficient mice (11). This manipulation allows for nearly complete reconstitution of immune responses to engrafted tumors.

Limitation of cancer cell lines, include adaptation to *in vitro* culture for extended periods of time and the selective pressure therein, which is not reverted during xenografting (12, 13), and the relatively minimal genetic diversity available in cell lines as compared to the tumors from which they derive (14, 15). To overcome some of these limitations, patient-derived xenografts (PDXs) are becoming more widely used (15). PDXs can be collected from patients early or late in their disease, as pre-treatment, responsive, or refractory to treatment (15). Recently, large therapeutic studies in solid tumor PDXs recapitulated response rates observed in clinical trials, highlighting the benefits of this model in translating data from the lab to the clinic (16).

**Figure 2:**
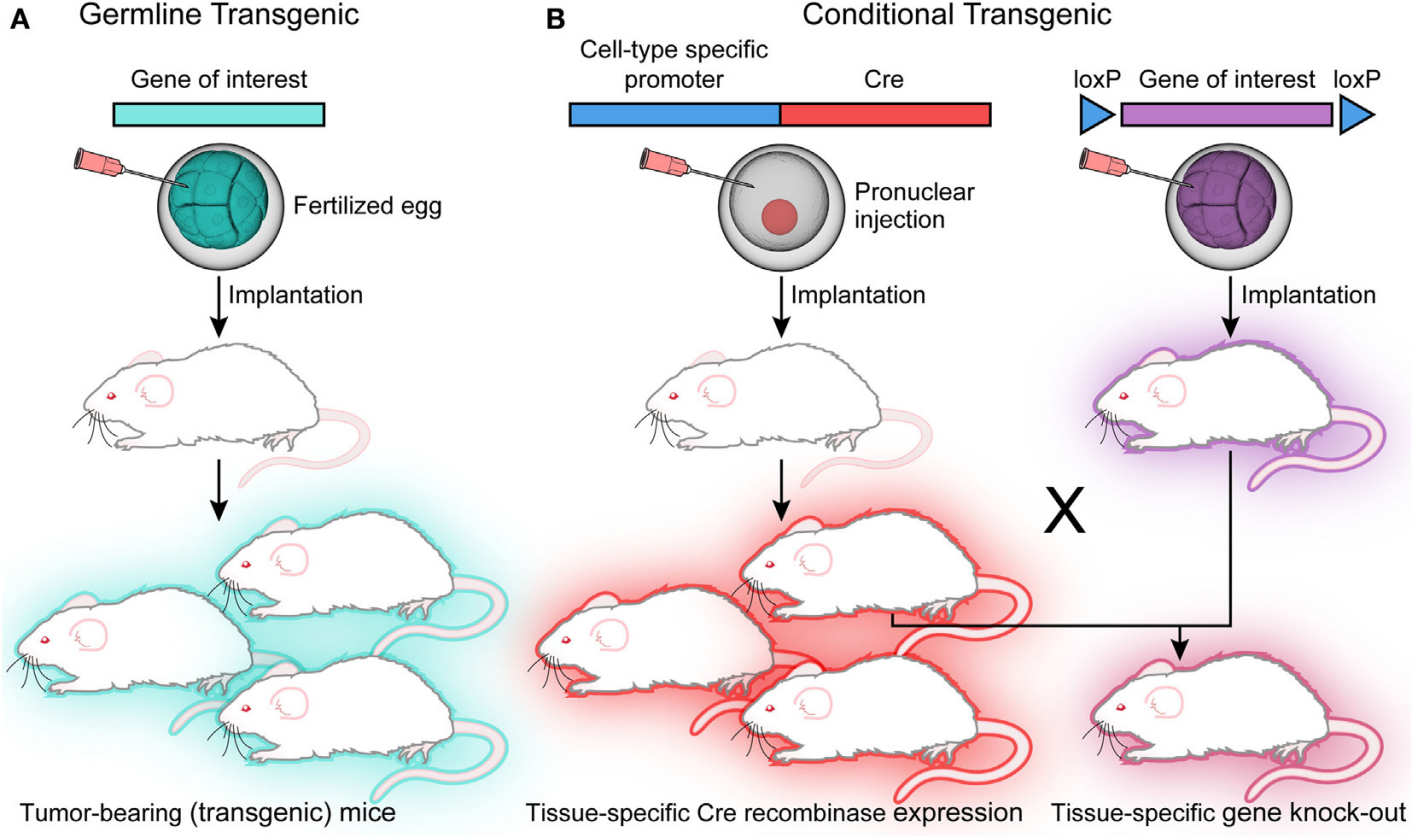
**Transgenic models**. **(A)** Injection of the vector construct into a fertilized egg will generate transgenic offspring that expresses the gene of interest in every cell. **(B)** Conditional transgenics can be generated for tissue-specific expression under the control of a Cre recombinase. These can be crossed with mice carrying the floxed gene of interest with resulting offspring carrying the tissue-specific knockout.

The genetically engineered mouse model (GEMM) has been used to mimic many human cancers with an etiology based in genetic aberration. To produce transgenic mice, a gene of interest in the form of a vector is injected into a fertilized egg. The resultant offspring will carry additional copies of this transgene (Figure 2). The gene of interest may also be engineered to express in a tissue-specific pattern or in response to drug treatment (5). Importantly, these tumors can be generated using immunocompetent mice, allowing investigation of both microenvironment and immunity on tumor development and growth. Therapeutic intervention can also be tailored to mimic clinical approaches, as either preventative or long-term therapy with the ability to follow-up tumor response *in vivo*.

Knockout models introduce loss of function mutations that disrupt gene function during embryogenesis, and knock-in models add an altered gene (5). One challenge with embryonic stem cell manipulation is the presence of genetic alteration during development, which may affect aberrant targets and inappropriately model an adult human disease. To avoid this undesirable effect, conditional models have been developed by crossing mice carrying recombinase effector genes with mice carrying the target gene, thus generating a spatially and temporally controlled mutation (5). There are available embryonic stem cell lines that carry floxed alleles of genes carrying Cre recombinase sites to allow transformation into blastocysts; wherein the mutation will be activated by crossing the mouse with the desired Cre recombinase driver (Figure 2) (5). Germline GEMMs allow research into mechanisms of oncogenic transformation, as well as the advantage of evaluating the efficacy of therapeutics on tumors with an intact microenvironment (4, 17). Inducible germline models, or knock-in systems, can be designed to activate mutations in specific tissues in response to drug or vector treatment (4, 18). While GEMMs are useful for evaluating the effects of a specific genetic alteration in tumor development, they are still not able to adequately reproduce the genetic complexity of human tumors (9).

Generation of murine models using clustered regularly interspaced short palindromic repeats (CRISPR)-Cas9 genome editing is an emerging and powerful tool to study cancer. CRISPR-Cas9 can be delivered directly into the mouse zygote, generating an animal carrying highly targeted genetic modification (19). This system creates double stranded DNA breaks in precise locations, which are then repaired by somewhat predictable mechanisms, and can produce null, conditional, single mutant, reporter, or tagged alleles in the mouse (20). Additionally, this system can be used to deliver combinations of guide RNAs to modify multiple genes in a single mouse hematopoietic stem cell, to more closely model the complexity of hematopoietic malignancy (21). Despite the risk of off-target editing noted in cell-based systems, recent data suggests that CRISPR-mediated editing events are accurate in the embryonic system (20, 22). It is likely that RNA-guided genome editing *in vivo* will become the preferred method of generating new and improved murine models.

A critical process in developing any murine model is phenotypic characterization. Phenotyping a mouse by a qualified comparative pathologist involves integration of the strain, background lesions, genetic manipulations by the researcher, the breeding regimen, the sex, diet, age, and any pathogenic or commensal organisms that may exist in the colony. Phenotyping often involves a combination of antemortem clinical assessments, postmortem pathological evaluation, and additional testing. Crossing various genotypes to develop a new paradigm warrants subsequent phenotypic characterization of the new line (6).

## Murine Models of Leukemia

Due to the many types of leukemia, this review will focus on the four major types of leukemia, accounting for 85% of all leukemias: acute and chronic myeloid leukemia (AML, CML), acute lymphoblastic leukemia (ALL), and chronic lymphocytic leukemia (CLL) (18). Incidence as well as survival rates have been steadily increasing, the latter due in large part to advancing targeted therapeutics (23). Murine models of leukemia have been critical in the understanding of leukemogenesis and the development of novel targeted therapy.

Of the spontaneous leukemic diseases, AML is the most intensively studied as it accounts for the majority of leukemia-related deaths (18, 23). Many of the genetic aberrations involved in the oncogenesis of AML have been characterized, with recurrent genetic abnormalities to include *CEBPA* mutations, *RUNX1* mutations, and *BCR-ABL1* gene translocations (24) (Table 2). Inherited mutations in numerous genes have also been identified in myeloid leukemia (24). Murine models have been instrumental in elucidating immune interaction, hematopoietic stem cell niche and microenvironment, cancer stem cells, novel therapeutics, and chemotherapeutic resistance in AML.

**Table 2:** **Murine models of leukemia**.

Disease	Gene targets	Model	Translation	Therapeutic use
AML	PU.1 + p53	Conditional knockout in hematopoietic cells	Aggressive AML	
	Nras:Bcl-2	Conditional transgenic	Myelodysplastic syndrome	Bcl-2 inhibitors
	TERC	Conditional knockout	Leukemia stem cell maintenance	
	AML-ETO	Inducible transgenic	APL	
	RARα fusion	Transgenic, variable	AML	Transretinoic acid

CML	BCR-ABL1	Humanized mice transplanted with retroviral vector	Chronic myeloproliferative syndrome	
		Conditional transgenic in hematopoietic cells	CML	Tyrosine kinase inhibitors
		Transposon-based insertional mutagenesis	Acute blast crisis	

Acute lymphoblastic leukemia (ALL)	ETV6–RUNX1	Transgenic using Ig heavy chain enhancer	Block in B-cell differentiation	
	E2A–PBX1	Conditional transgenic using Lck enhancer, TCR Vβ promoter	B-cell ALL	
	NOTCH1	Tumor-derived engraftment of NOD/SCID	Xenograft T-ALL	Monoclonal antibody against Notch1
	PRDM14	Inducible transgenic	Rapid onset T-ALL	Monoclonal antibody against Notch1

Chronic lymphocytic leukemia (CLL)	miR-16	Spontaneous in New Zealand Black	Clonal CD5+ B cell disease	
	T-cell leukemia 1	Serial transfer transgenic	Rapid progression CLL	PD-1 immune checkpoint inhibitor
	BCR	NSG™ with orthotopic splenic engraftment	CLL	Ibrutinib efficacy

Homozygous deletion of the upstream regulatory element of *PU.1* results in downregulation of this protein within hematopoietic stem and progenitor cells and leukemia development in a mouse model of AML (25). Deletion of *Tp53* in these mice results in a more aggressive disease with shortened survival. This model was useful in identifying Myb and miR-155 as contributing to *PU.1* downregulation (25). An attractive model of myelodysplastic syndrome transformation to AML is a transgenic model featuring N-Ras/Bcl-2 mitochondrial complex inducing disease progression (26). This model has been used to study the therapeutic efficacy of Bcl-2 inhibitors for the treatment of AML and other leukemic diseases (26).

Genetic deletion of the telomerase subunit Terc in a retroviral-induced AML mouse model results in cell-cycle arrest and apoptosis of leukemia stem cells (LSCs) which maintain AML (27). The LSCs of these *TERC* (−/−) mice express a similar gene expression profile as human AML patients with better survival following chemotherapy (27). Inhibition of telomerase in a xenograft model shows specific targeting of LSCs with reduced disease progression and delayed relapse, identifying telomerase as an attractive therapeutic target (27).

CML is characterized by the malignant transformation of hematopoietic stem cells, predominantly as a result of dysregulated signaling through tyrosine kinases (28). CML eventually progresses to a fatal myeloblastic phase with an accumulation of immature hematopoietic cells following inactivation of tumor suppressors and differentiation factors (29). A reciprocal translocation between chromosomes 9 and 22 (*t*(9;22)–(q34;q11)), resulting in fusion protein BCR–ABL1 is a major contributing event to CML and has been a dominant therapeutic target (30, 31) (Table 3). The original description of the murine model by Daley et al. revealed induction of a chronic myeloproliferative syndrome in irradiated mice transplanted with a retroviral vector encoding the BCR–ABL1 fusion protein that closely resembled human CML (30). Other approaches have included xenografts with patient cells or cell lines, retroviral transduction of bone marrow-derived cells followed by transplantation into irradiated congenic mice, and transgenic mice expressing oncogenic BCR–ABL1 (28).

**Table 3:** **Developed murine models featuring expression of oncogenic fusion proteins**.

Disease	Fusion protein	Model	Reference
AML	BCR–ABL1	NSG xenograft with MSC scaffold	(32)
	Mixed-lineage leukemia–AF9	NSG xenograft with MSC scaffold	(32)
	AML1–ETO	Irradiated C57BL/6J with intravenous autologous transfected BM cells	(33)
CML	BCR–ABL1	BCR-ABL retrovirus co-expressing GFP in a triple gene system	(34)
ALL	ETV6–RUNX1	Inter-cross ETV6–RUNX1 and Pax5 heterogeneic mice	(35)
	E2A–PBX1	Conditional transgenic E2A–PBX1 under the control of Mb1 or Mx1 promoter-Cre	(36)
Peripheral T-cell lymphoma	ITK–SYK	ITK-SYK cloned into ROSA26 targeting vector, crossed to CD4-Cre	(37)

*NSG, NOD/SCID IL2Rγ−/− mouse; MSC, mesenchymal stem cell; BM, bone marrow*.

Murine models utilizing retroviral vectors allowed identification of regions within the BCR–ABL1 fusion protein that are critical for transformation, allowing for the rational design and development of tyrosine kinase inhibitors (30). Murine models have been used to recapitulate the different clinical phenotypes noted in patients with various BCR–ABL1 fusion proteins (38). Retroviral murine models also allow functional evaluation of individual genes concerning CML development and progression, including the finding that STAT5 expression is necessary for BCR–ABL1-mediated leukemogenesis (39, 40). In humanized models, retroviral expression in cord blood followed by transplantation to immune-deficient mice resulted in an accumulation of pre-B-cells, a differentiation block that has been seen in patient cells (41).

Non-conditional models have been successful at mimicking the clinical characteristics of human patients (42). Particularly, use of separate strains of mice to generate inducible models allows avoidance of early gene expression (43, 44). Newer transgenic models allow expression of the fusion protein exclusively within the hematopoietic stem cell compartment (45). While the chronic phase of CML is dependent on BCR–ABL1, progression to acute blast crisis is mediated by additional genetic alterations, and murine models of this disease progression are necessary to develop therapies for this patient subset who are unresponsive the tyrosine kinase inhibitors (46). A murine model using transposon-based insertional mutagenesis in a background of chronic CML elucidated a unique pattern of insertions thus identifying candidate genes for the pathogenesis of blast crisis (46). Thus, murine models were not only instrumental in the identification of the mechanisms of leukemogenesis but also have contributed to advances in understanding disease progression in CML and identification of novel therapeutic targets.

Acute lymphoblastic leukemia is a disease of childhood and older adults, in which many genetic alterations have been identified as potential drivers of leukemogenesis. There are T-cell and B-cell variants of ALL, each having differing pathogeneses. Systems used to study ALL have included syngeneic models and xenografts, which have inherent limitations in that they cannot model the entirety of host and microenvironment contributions to leukemia development (47). Syngeneic models utilize genetically modified primary cells followed by transplantation, as well as the development of transgenic models that alter gene expression in lymphoid cells (47).

Chromosomal translocation resulting in fusion protein ETV6–RUNX1 is the most frequent rearrangement in pediatric ALL (48), and its expression correlates with a good prognosis (49). Retroviral transduction of this fusion protein does not result in leukemia in several models but instead leads to a block in B-cell differentiation (50). Neither did leukemia develop in a transgenic model utilizing the immunoglobulin heavy chain enhancer to drive ETV6–RUNX1 expression (51). Another fusion protein in ALL, E2A–PBX1, was expressed under lymphoid-specific Lck enhancer and the TCR Vβ promoter to successfully cause B-cell ALL when crossed with CD3ε−/− mice (52). The most frequent rearrangements occur with mixed-lineage leukemia, conferring a worse prognosis (53). Murine models to study many of these fusion proteins have had mixed results with regards to the development of ALL mimicking the human disease (47).

NOTCH1 activity is increased in a significant population of patients with T-ALL secondary to Notch1 mutations or alterations in FBW7 gene (54). Pediatric T-ALL was engrafted in NOD/SCID mice to test responses to a novel monoclonal antibody against NOTCH1 (55). This antibody delayed engraftment in T-ALL samples with Notch1 mutations, even in samples derived from patients who were poorly responsive to previous therapy (55). Inducible overexpression of PRDM14, a pluripotency maintenance gene for embryonic stem cells, in a Cre recombinase system in mice induces rapid onset highly penetrant T-ALL which also features high Notch1 activity with high expression of NOTCH1 downstream targets. The T-ALL cells from this model are also sensitive to NOTCH1 inhibitor therapy (56). Interestingly, overexpression of IL-15 in a transgenic mouse model produces large granular cell leukemia with a NK/T cell phenotype (57).

Chronic lymphocytic leukemia is the most common form of leukemia in the United States and is characterized by proliferation of CD5+ B cells in bone marrow, peripheral blood, and lymphoid tissues (58). A common genomic aberration in CLL leads to increased expression of anti-apoptotic protein BCL-2, which is negatively regulated by miR-15a and miR-16-1. The expression of these miRs is lost *via* deletion of a region on chromosome 13, 13q14.3 (59, 60). Both of these miRs decrease Bcl-2 expression by posttranscriptional regulation resulting in induction of apoptosis (61). New Zealand Black mice develop a spontaneous clonal CD5+ B cell disease in old age, similar to a subtype of CLL in humans. This disease has been linked to a locus on murine chromosome 14 with synteny to the human chromosomal locus lost in CLL. These mice also have reduced expression of miR-16-1 (62). Development of CLL in these mice is accelerated by induction of a heterozygous mutation in *IRF4* (63).

The proto-oncogene T-cell leukemia 1 (TCL-1) is expressed in pre-B cells and early T-cell precursors. Overexpression of TCL-1 has been identified in multiple B-cell lymphomas and in the majority of CLL patients (64). The TCL-1 transgenic mouse mimics human CLL; however, disease development is delayed leading to practical issues with experimentation and therapeutic investigation (65). Serial transfer in TCL-1 transgenic mice allows rapid progression of the disease and has been useful for preclinical studies (64). One such study examined the efficacy of programmed cell death (PD-1) immune checkpoint inhibitors, an exciting therapeutic target for CLL (66). Additionally, crossing the TCL-1 mice with other GEMMs has aided in elucidating the role of other survival factors in CLL, such as *ROR1* and *BAFF* (64). To model a common genetic alteration in the human disease, a transgenic mouse lacking the chromosomal region 13q14 encoding for *DLEU-2*, miR-15, and miR-16 were developed (67). This mouse demonstrated a spectrum of lymphoproliferative disorders including a progressive CLL; however, the penetrance was poor (65).

Poor engraftment by CLL cells into mice has limited the utility of xenograft models for this disease. Engraftment models are improved in NOD/SCID mice, which have been used to characterize prognostic biomarkers (68). CLL cells depend on signaling from the microenvironment, and the use of NSG™ mice was then a great benefit to study microenvironment contribution to disease development (69, 70). NSG™ mice were also used to demonstrate that the murine splenic microenvironment supported CLL cell proliferation to a similar degree as human lymph nodes with induction of BCR and NF-κB pathways. This model was also used to study the effects of ibrutinib on the microenvironment and tumor burden (71). Importantly, a CLL cell line was recently established from a patient and was maintained in coculture with autologous stromal cells. This line was readily transplantable into NSG™ mice that developed the multi-systemic disease (72).

Infection with human T-cell leukemia virus-1 (HTLV-1) causes adult T-cell leukemia (ATL) in a minority of infected people. Murine models have been used to study persistent viral infection and tumorigenesis induced by HTLV-1 protein expression through the use of transgenics, xenografts, and infection of humanized mice with the virus (73). Overexpression of oncogenic Tax and HBZ viral proteins from HTLV-1 has elucidated mechanisms of leukemia development. Engraftment of ATL cell lines into SCID mice has been used to study tumor spread and metastasis, as well as for evaluation of novel therapeutics. Finally, humanizing mice with CD34+ cord stem cells and subsequently infecting them with HTLV-1 leads to leukemia development (73).

## Murine Models of Lymphoma

B-cell lymphomas are the fourth most common hematologic malignancy in humans and the most common type of non-Hodgkin’s lymphoma (74). The most common B-cell lymphomas are diffuse large B-cell lymphoma (DLBCL), follicular lymphomas (FLs), marginal zone lymphomas, and Burkitt’s lymphoma (BL) (74). Murine models of lymphoma have allowed the study of tumor biology, microenvironment, and mechanisms of response to therapy.

Translocation of the *MYC* oncogene to a site downstream of a B-cell specific enhancer or promoter region results in B-cell lymphoma. The transgenic murine model Eμ-MYC features *MYC* gene insertion into the IgH locus with a 100% incidence of B-cell lymphoma developing (74) (Table 4). This model features development of an immature form resembling BL and a more indolent mature form resembling DLBCL (75). Modification of similar models by an introduction of murine retroviruses expressing ras oncogene can be used to produce accelerated lymphomagenesis (76).

**Table 4:** **Murine models of lymphoma**.

Disease	Gene targets	Model	Translation	Therapeutic use
B-cell lymphoma	MYC	Conditional transgenic using Ig heavy chain	B-cell lymphoma, Burkitt’s lymphoma, diffuse large B-cell lymphoma	
	MYC + RAS	Conditional transgenic with retrovirus transduction	Accelerated development of B-cell lymphoma	CD20 immunotherapy
	SYK	MYC/BCR/sHEL transgenic	B-cell lymphoma	SYK inhibitors

Follicular lymphoma (FL)	BCL-2	Transgenic linked to Vav regulatory sequence	FL	

EBV-induced disease		Humanized mice infected with EBV	B-cell lymphoproliferative disease, hemophagocytic lymphohistiocytosis	

Peripheral T-cell lymphoma (PTCL)	ITK-SYK	Inducible transgenic using CD4-Cre	Disseminated PTCL	SYK inhibitors

Anaplastic large cell lymphoma (ALCL)	NPM-ALK	Inducible transgenic using CD4-Cre	ALCL	

Cutaneous T-cell lymphoma (CTCL)	IL-15	Transgenic	CTCL	HDAC inhibitors

Follicular lymphoma is the second most common nodal lymphoma and progresses slowly with generally favorable response to therapy, but development of resistance is a common clinical problem (77). At (14:18) translocation in FL activates Bcl-2 expression by linking it to the IgH locus. Use of the Vav gene regulatory sequences to drive Bcl-2 expression in mice results in the development of FL (78). This model was used to study the role of prolonged germinal center reactivity and V-gene hypermutation. Embryonic deletion of the activation-induced cytidine deaminase (AID) gene in mice prevented Bcl-6-driven FL, suggesting the important role of *AID* in the generation of additional genetic alterations in the pathogenesis of this disease (79). Spleen tyrosine kinase (SYK) was shown to be required for survival of non-Hodgkin lymphoma-like tumors in an Eμ-MYC/BCR/sHEL-transgenic mouse. A specific inhibitor of Syk was used to cause tumor regression *in vivo* (80). One such study further identified galectin-1, a carbohydrate-binding protein with diverse functions in immune response, as a key determinant in the development of resistance to antibody therapy (81).

Humanized mice infected with Epstein–Barr virus develop a B-cell lymphoproliferative disease and EBV-associated hemophagocytic lymphohistiocytosis. The use of these models has also elucidated the role in innate immune responses including EBV-specific adaptive T-cell responses in these diseases (82). EBV-associated Hodgkin’s lymphomas developed in mice with activated T-cell environment, while non-Hodgkin’s lymphoma developed in T-cell depressed mice following infection with EBV (83). Further, EBV mutants with altered latency genes have been used to generate aggressive lymphoproliferative disease in mice, allowing the better understanding of the roles of these genes in disease pathogenesis (82).

Peripheral T-cell lymphoma (PTCL) is a rare and aggressive form of non-Hodgkin lymphoma that responds poorly to standard chemotherapeutic treatment. A translocation was identified in a subset of PTCL patients featuring a fusion of *ITK* and *SYK* (84). Subsequently, a mouse model was developed using a loxP stop cassette to generate an inducible fusion protein crossed with CD4-Cre animals (85). The resulting transgenic mouse expressed the kinase fusion protein in T-cells and developed lymphoma mimicking disseminated PTCL. Additionally, this mouse demonstrated responsiveness to Syk inhibitors (85). A model of anaplastic large cell lymphoma, a PTCL of young adults, utilizes CD4/NPM-ALK transgenic mice with increased NOTCH1 expression (86).

Cutaneous lymphomas are a heterogeneous group of non-Hodgkin lymphoma that primarily affects the skin. While xenograft models are in use for therapeutic efficacy studies in cutaneous lymphoma, a model to study the development and progression of this rare disease has been lacking (87). Cutaneous T-cell lymphoma (CTCL) is the most typical of cutaneous lymphomas. A method of intrahepatic injection of CTCL-derived cell lines into NOD/SCID/IL2rγ mice resulted in successful engraftment and had been used to evaluate cell line tumorigenicity as well as therapeutic responses in preclinical studies (88). Recently, a transgenic mouse model with constitutive global overexpression of pro-inflammatory cytokine IL-15 was described, which develops a infiltration of the skin by mature T-cells characterized by the same immunophenotypic features as found in the human disease (89). Thus this model will be invaluable in investigations of lymphomagenesis in CTCL as well as clinical response to therapies with activity in a subset of human CTCL.

## Discussion

Development of translational murine models has been vital to the detailed mechanistic investigation into disease pathogenesis and identification of therapeutic targets. The mouse provides a valuable model system to study human disease in its genetic and physiologic similarity while being readily available and economically practical.

One needs to look no further than the failure rate of newly developed therapeutics to understand the importance of appropriate interpretation of mouse data. While murine models are frequently used for preclinical investigations of novel drug targets, success in these studies is often poorly predictive of success in the clinic. Indeed, the average rate of successful advancement from animal models to clinical trials for cancer drugs is less than 8% (90). Of compounds that are successful in preclinical modeling, 85% of early human trials fail (90). This attrition rate can be improved by careful consideration, selection, and design of murine models (91, 92). Unfortunately, there are currently no best-practice standards for animal testing, and study design practices are variable.

In this review, the following considerations for a researcher designing and utilizing a murine model have been emphasized: is the mouse the most appropriate *in vivo* model? What are the background lesions or strain characteristics that could confound my study? Am I expressing my gene of interest in the right cell type context and at the right time in disease development? Is the model disease similar genetically, morphologically, and phenotypically to the human condition? Ultimately, the usefulness of the model must be tempered by its shortcomings, and therefore, the use of murine models must be approached with a thorough understanding of both its utility and limitations.

One final question, Is there a better way? Phase “0” studies utilize miniscule doses of novel drugs in human subjects to collect data on pharmacodynamics and target specificity (90, 93). An exciting alternative to animal studies in preclinical research is the “organ on a chip” methodology which recapitulates the organ structure, microenvironment, and physiological function by implanting the organ onto silicon chips (90, 94). This technology may allow faster and less expensive drug development with the ability to successfully and faithfully mimic disease states on a chip. Thus far, this technology has not been used to address the pathology of liquid tumors. However, the field is quickly advancing and, may shortly, allow *in vitro* examination of hematopoietic systems and hematopoietic neoplasia.

Although the mouse’s reign as king in biomedical research will likely continue for many years, appropriate model design, thorough characterization and excellent new technologies will allow researchers to strive further to replace, reduce, and refine their use.

## Author Contributions

RK, PP, and AM contributed to writing and editing the material presented in the article.

## Conflict of Interest Statement

The authors declare that the research was conducted in the absence of any commercial or financial relationships that could be construed as a potential conflict of interest.
